# A script to highlight hydrophobicity and charge on protein surfaces

**DOI:** 10.3389/fmolb.2015.00056

**Published:** 2015-10-13

**Authors:** Dominique Hagemans, Ianthe A. E. M. van Belzen, Tania Morán Luengo, Stefan G. D. Rüdiger

**Affiliations:** Cellular Protein Chemistry, Bijvoet Center for Biomolecular Research, Utrecht UniversityUtrecht, Netherlands

**Keywords:** protein-protein interaction, surface hydrophobicity, charge pairs, PyMOL, amino acid properties

## Abstract

The composition of protein surfaces determines both affinity and specificity of protein-protein interactions. Matching of hydrophobic contacts and charged groups on both sites of the interface are crucial to ensure specificity. Here, we propose a highlighting scheme, YRB, which highlights both hydrophobicity and charge in protein structures. YRB highlighting visualizes hydrophobicity by highlighting all carbon atoms that are not bound to nitrogen and oxygen atoms. The charged oxygens of glutamate and aspartate are highlighted red and the charged nitrogens of arginine and lysine are highlighted blue. For a set of representative examples, we demonstrate that YRB highlighting intuitively visualizes segments on protein surfaces that contribute to specificity in protein-protein interfaces, including Hsp90/co-chaperone complexes, the SNARE complex and a transmembrane domain. We provide YRB highlighting in form of a script that runs using the software PyMOL.

## Introduction

Protein-protein interactions underlie all processes in the cell. Specificity of protein-protein interactions is determined by matching of complementary functional groups with those of the opposite surface (Chothia and Janin, 1975; Eaton et al., 1995). Adequate highlighting of protein surfaces in structural models allows identification of specificity determinants. Widely used highlighting schemes include CPK coloring and highlighting according to electrostatic potential or hydrophobicity gradient. CPK coloring highlights atoms per type and allows distinguishing between different elements in a protein (Corey and Pauling, 1953; Koltun, 1965). Electrostatic potential maps highlight protein surfaces based on the Boltzmann equation, representing a gradient from negative to positive potential. Hydrophobicity is frequently visualized by a hydrophobic to non-hydrophobic color gradient based on experimentally based classification of amino acid properties (Eisenberg et al., 1984).

Assessment of major determinants for protein-protein interactions typically requires a combination of the aforementioned color schemes. It would be helpful for assessing protein interaction surfaces, e.g., for planning mutations, to highlight the major determinants for protein interactions in a single image. Such a color scheme should also indicate protein properties at atomic level, to account for differences in chemical properties within side chains.

Here, we present a scheme to color proteins by charge and hydrophobicity at atomic level. The YRB scheme specifically highlights all carbon atoms that have high potential to form hydrophobic interactions in yellow. Simultaneously, nitrogen atoms in the side chain of arginine and lysine are colored blue and oxygen atoms in the side chains of glutamate and aspartate are red. This visualizes complementarity in functional groups, such as hydrophobic and charged groups, as we illustrate in interaction of the Hsp90 chaperone with its partner proteins and within the interface of the SNARE complex. The YRB scheme can be used universally for a protein of interest when loaded into the visualization platform PyMOL. The script facilitates a quick assessment of protein surfaces and visualizes specificity in protein-protein interfaces.

## Materials and methods

### PyMOL for molecular visualization

To apply YRB highlighting, we used PyMOL [The PyMOL Molecular Graphics System, Delano Scientific, San Carlos, CA, USA, version 1.4 (Mac OS X) and version 0.99 (Windows)]. PyMOL is a widely used open-source molecular visualization platform, which can be extended by plugins and scripts written in Python (Python Software Foundation. Python Language Reference, version 2.7, released on July 3rd 2010), available at http://www.python.org.

### Highlighting atoms according to charge and hydrophobicity

**Figure 1:**
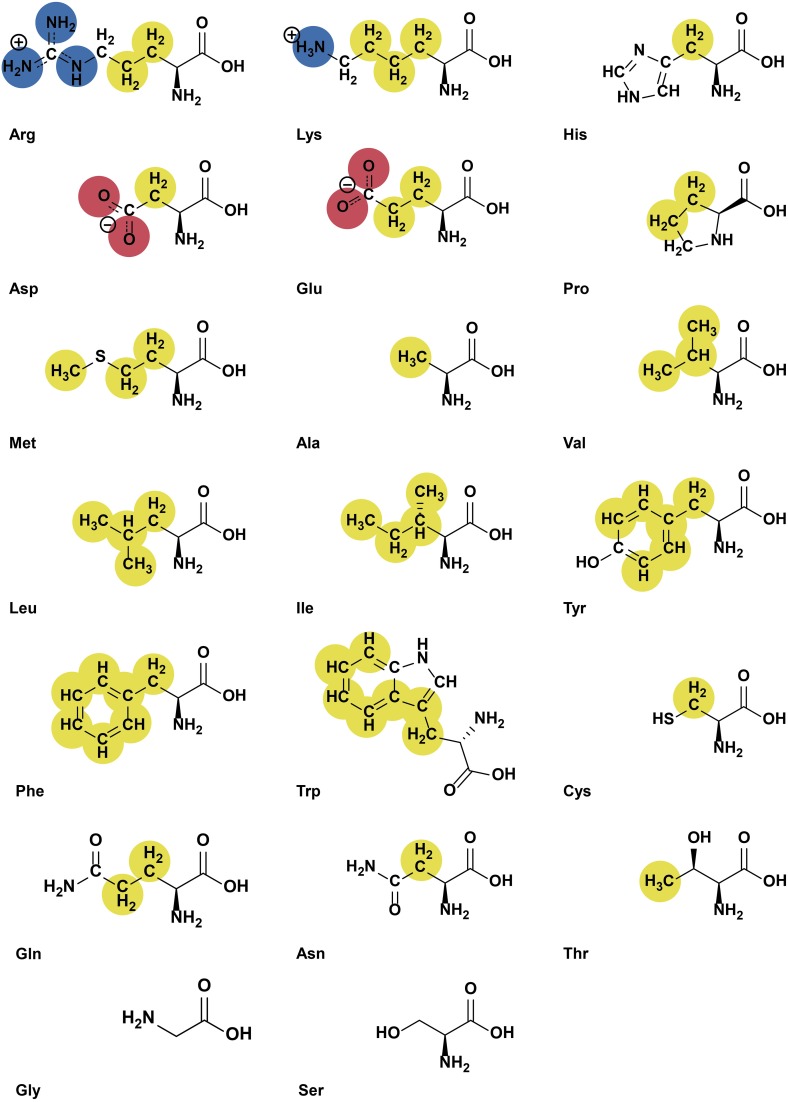
**Highlighting at atomic level visualizes both hydrophobicity and charge**. Functional groups are colored according to the YRB highlighting scheme (hydrocarbon groups without polar substitutions, yellow; negatively charged oxygens of glutamate and aspartate, red; nitrogens of positively charged functional groups of lysine and arginine, blue; all remaining atoms including the polar backbone, white).

The YRB script colors structural models of proteins at atomic level. Carbon atoms not bound to nitrogen or oxygen atoms are colored yellow, oxygens carrying the negative charges in glutamate and aspartate red and nitrogens carrying the positive charges in lysine and arginine blue, while all remaining atoms are white (Figure 1). The coloring reflects the typical charges at physiological pH and does not consider the potential different chemical environment in some active centers. Therefore, histidine is always represented uncharged. The source code of the Python script is provided in the Supplementary Data.

### Running the YRB script in PyMOL

The YRB script is loaded into PyMOL by selecting “*File: Run.”* When the structure of interest is also loaded into PyMOL, the script is run by the command “*yrb*” in the command screen. This will color the structures according to the YRB highlighting scheme. If multiple structures are loaded into PyMOL, a specific structure can be selected for highlighting by the command “*yrb* <*designation*>,” in which “<designation>” is to be replaced with the actual name of the structure. Proteins often have their PDB code as designation, but any designation can be given to a selection by using the PyMOL command “*create.”*

### Python script applies YRB highlighting to proteins

When the script is run in PyMOL, hydrogen atoms are removed and the exact colors are defined with RGB color values. A hash table is used to map amino acids to a list of ordered pairs of atoms with their color according to their position in the side chain and which atoms they are bound to (Figure 1).

The script considers all molecules present in PyMOL individually. If the designation of the structure matches the given command, the script proceeds to highlighting. When no specifications are made by the user then all molecules will be highlighted.

**Figure 2:**
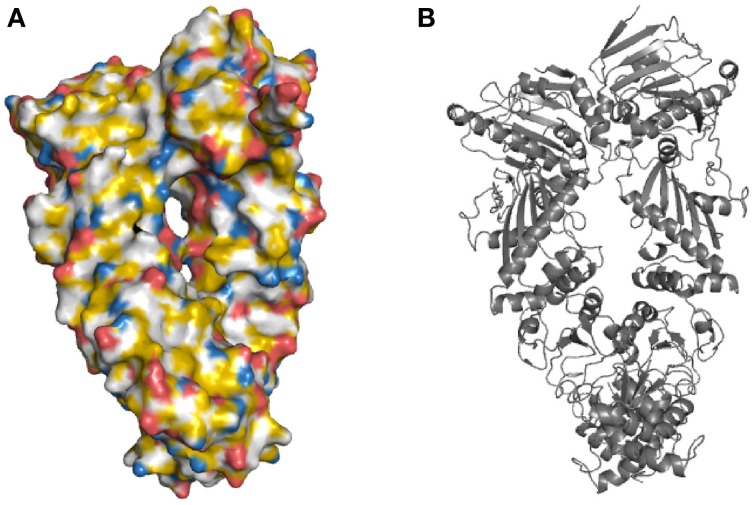
**The yeast Hsp90 surface highlighted in YRB. (A)** Hsp90 highlighted according to the YRB highlighting scheme or, **(B)** displayed in cartoon. Yeast Hsp90 (A2-N676) PDB code 2cg9 (Ali et al., 2006).

First, the backbone atoms N, C, CA, and O of all amino acids are colored white. CB atoms that are not bound to nitrogen or oxygen atoms are colored yellow, while the oxygen-bound CB of serine and threonine remain white. Then the remaining atoms of the amino acid side chains are colored. The side chains are considered one at time when the script runs through the mapping and every single atom of the side chains is colored accordingly. For example, the CG atom of arginine is bound to carbon atoms and becomes yellow. The nitrogen atoms, NE, NH1 and NH2 of arginine are colored blue. This results in the molecule being colored in yellow, red, blue, and white at atomic resolution (Figure 2).

### Analysis of alternative highlighting schemes

All alternative color schemes were applied using PyMOL. The electrostatic potential maps were applied on protein surface by the option “*vacuum electrostatics.*” The convention of chemical elements was applied by the option “*color by elements.*” The red to white hydrophobicity gradients were applied by running the script “*color_h”* on the molecule in question (Eisenberg et al., 1984).

### Step-by step protocol for applying YRB to any protein of interest

1. Install both PyMOL and Python on your computer (https://www.python.org/).
2. Save “The YRB script” at a desirable location, either with .py (Windows) or .pym (MacOS X) extension.
3. Find the pdb file of interest in the Protein Data Bank.
4. Open the pdb file into PyMOL or type “*fetch*<*pdbcode*>” (connection to the internet is needed).
5. The surface representation is created by either typing “hide everything; show surface” or clicking on “s: show as surface” in the right-hand panel.
6. The YRB scheme is loaded onto the surface by clicking in the menu: “File: Run.”
7. Open the YRB file from the location where it was saved before.
8. The YRB script is applied by typing: “yrb.”

Additional steps are needed to display the interaction interface in YRB colors. Amino acids that are not involved in the interaction can be colored gray.

(9) Make the amino acid sequence visible by clicking on “*s*” in the bottom right corner.
(10) Select the amino acids that are not involved in the interaction in the sequence window above the protein.

Color this selection *(sele)* gray, so that only the interaction interface is colored in YRB.

(11) Next to the selection “*(sele)”* the color can be adapted: by selecting “*c: color grays: gray50*” in the upper right corner.

## Results

### YRB scheme highlights atoms according to their atomic properties

We set out to use a highlighting scheme that combines several features that facilitate assessment of protein interaction interfaces considering the following features: (i) It should highlight surface properties at atomic level (ii) It should visualize hydrocarbon groups (CH_n_) with non-polar substitutions but not those with polar ones. (iii) It should visualize the charged groups in interaction interfaces. (iv) It should be limited to using primary colors to effectively highlight surface properties in an intuitive way.

To visualize hydrophobic and charge contributions to protein interfaces we use the following highlighting scheme at atomic level using the colors yellow, red, and blue (YRB). In this scheme, all carbon atoms not bound to nitrogen and oxygen atoms are highlighted in yellow, nitrogen atoms in the side chains of lysine and arginine are blue, oxygen atoms in the side chains of glutamate and aspartate are red and all remaining atoms white (Figure 1).

This color scheme reflects the key points that are relevant for intuitive assessment of protein-protein interactions: (i) Amino acids are composed of atoms with different properties. (ii) CH_n_ groups that are not bound to electronegative oxygen or nitrogen have a high potential for hydrophobic interactions. (iii) Charged pairs play a key role in protein-protein interactions. (iv) The YRB scheme combines hydrophobic and charged functional groups, which reflects specificity in protein-protein interfaces.

The benefit of highlighting at atomic level is particularly evident for residues with polar or charged groups. The side chains of polar and charged amino acids contain non-polar segments, which may contribute to the hydrophobic interface. They are, however, unable to engage in coulomb and polar-driven interactions, as would be suggested if the protein is highlighted at residue level. Therefore, YRB colors e.g., the β-carbon of arginine yellow and the nitrogen atoms of its positively charged head group blue (Figure 1). Also in case of polar residues, only carbon atoms that are not functionalized with oxygen or nitrogen are highlighted yellow. For instance, in case of threonine, the γ-carbon is highlighted yellow but the β-carbon is not, as this atom is bound to a polar hydroxyl group (Figure 1).

### Interfaces highlighted in YRB visualize complementarity

**Figure 3:**
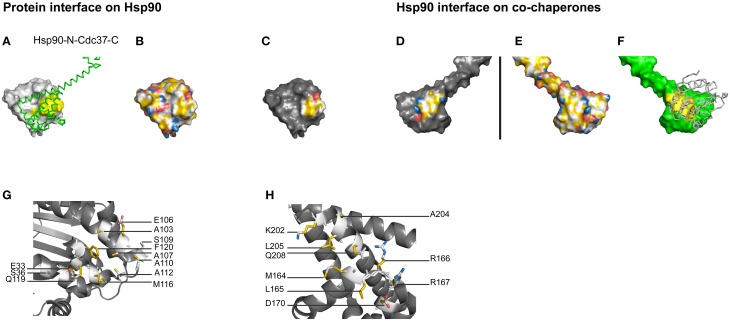
**Hydrophobic core of the Hsp90-Cdc37 interface**. **(A–H)** Complex of yeast Hsp90-N (A2-L207) and Cdc37C (H148-K347), PDB code 1us7 (Roe et al., 2004). **(C,D)** Complementary views of the interfaces between Hsp90 and the mirrored co-chaperones are displayed in YRB. **(B,E)** Full YRB highlighting on both proteins. **(A,F)** The complementary interface (yellow) of Hsp90 (gray) and co-chaperones (green) are either shown in ribbon or in surface. **(G,H)** Hsp90-Cdc37 interface displayed in YRB stick mode. The charged pair E33 of Hsp90 and R167 of Cdc37 is visible in sticks mode.

**Figure 4:**
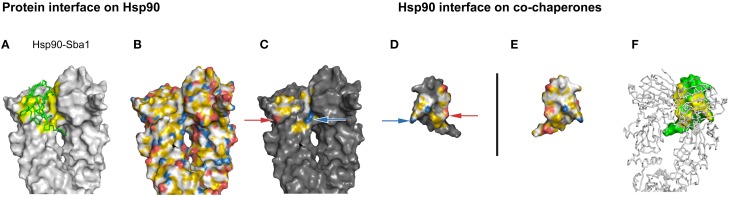
**Complementarity in the YRB highlighted Hsp90-p23 interface**. **(A–F)** Complex of yeast Hsp90 (A2-N676) and Sba1 (W12-A135), PDB code 2cg9 (Ali et al., 2006). **(C,D)** Complementary views of the interfaces between Hsp90 and the mirrored co-chaperone p23 are displayed in YRB. The matching ion pairs K27 with D122 and D113 with K113 of the Hsp90-p23 interface are indicated by arrows. **(B,E)** Full YRB highlighting on both proteins. **(A,F)** The complementary interface (yellow) of Hsp90 (gray) and p23 (green) are either shown in ribbon or in surface in the first and last panels.

**Figure 5:**
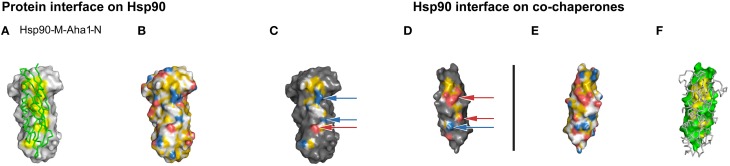
**Complementarity in the YRB highlighted Hsp90-Aha1 interface**. **(A–F)** Complex of yeast Hsp90-M (T273-D527) and Aha1-N (W11-D153), PDB code 1usv (Meyer et al., 2004). **(C,D)** Complementary views of the interfaces between the fragments of Hsp90 and the mirrored co-chaperone Aha1 are displayed in YRB. The ion pairs of the Hsp90-Aha1 interface K390 with D68, K514 with D110, and E515 with R128 are indicated by arrows. **(B,E)** Full YRB highlighting on both proteins. **(A,F)** The complementary interface (yellow) of Hsp90 (gray) and co-chaperone (green) are either shown in ribbon or in surface in the first and last panels.

To test the potential of YRB highlighting in the understanding of protein-protein interfaces, we analyzed protein complexes with known crystal structures. A particularly suitable system is the ATP-dependent molecular chaperone Hsp90, which interacts with a plethora of co-factors that regulate its functional cycle (Pearl and Prodromou, 2006; Li et al., 2012). For several of them, co-crystal structures are available that allow studying the binding interface. Here, we analyse the interaction of Hsp90 with the substrate targeting factor Cdc37 and the ATP cycle regulating co-chaperones p23 and Aha1 to assess the potential of YRB highlighting (Figures 3–**5**).

Cdc37 interacts with the N-terminal domain of Hsp90 (Hsp90-N), preventing its dimerisation (Roe et al., 2004; Siligardi et al., 2004; Vaughan et al., 2006). The crystal structure of Cdc37 and Hsp90-N reveals that this interface consists of hydrophobic interactions surrounded by seven hydrogen bonds and one charged pair (Supplementary Table 1; Roe et al., 2004). The single ion pair in the Hsp90-Cdc37 interface is not explicitly visible in the YRB surface representation, as the negatively charged residue in Hsp90 is not entirely exposed to the surface. YRB highlighting does represent the Hsp90-N-Cdc37 interface with a hydrophobic center surrounded by polar and charged groups (Figures 3C,D). This pattern is complementary in both the Hsp90 and the Cdc37 side of the complex interface. Thus, YRB highlighting primarily reveals the hydrophobic core and in addition a ring of charged groups surrounding the hydrophobic core that matches with the opposite surface (Roe et al., 2004).

Now, we set out to investigate the interface of Hsp90-p23 to analyze whether hydrophobic interactions in combination with charged pairs are also represented by YRB. The interaction of the co-chaperone p23 with Hsp90 has been revealed in a crystal structure of the yeast homologs of a complex consisting of the folded part of p23 and all three domains of Hsp90 (Supplementary Table 2; Ali et al., 2006). The p23 protein interacts with the middle domain of Hsp90 (Hsp90-M) and in between the two N-terminal domains of the Hsp90 dimer. The interface that is positioned in the Hsp90-M domain consists of one ionic bridge and several hydrophobic interactions. The interface between p23 and the two N-termini of Hsp90 consists of hydrophobic interactions, one charged pair and some polar interactions (Ali et al., 2006). The Hsp90-p23 interface highlighted in YRB displays the hydrophobic interaction in yellow and the two charge-charge interactions that match in complementary views (Figures 4C,D). Thus, an interface with both charged and hydrophobic interactions is displayed in YRB.

To test whether YRB also represents an interface that contains many charged pairs, we colored Hsp90-Aha1 interface according to the YRB scheme. The cochaperone Aha1 activates the ATPase activity by interacting with the middle domain of Hsp90 and partially overlaps with the p23 interface in Hsp90. The core of the Hsp90-Aha1 interface primarily consists of a hydrophobic patch supported by seven charged pairs (Supplementary Table 3; Meyer et al., 2004). YRB highlighting visualized both the hydrophobic patch and the matching charged pairs on both sides of the interface (Figures 5C,D). Thus, YRB adequately represents an interface that is dominated by electrostatic interactions. Together, we conclude that the YRB highlighted interfaces visualize complementarity in both hydrophobicity and charge.

### Hydrophobic interactions elucidated in YRB

**Figure 6:**
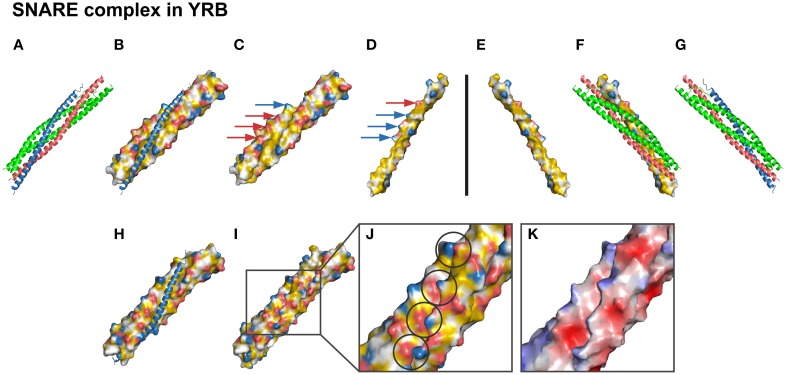
**YRB highlights specificity of the SNARE interface**. **(A–K)** Complementary views of the interface between t-SNAREs and v-SNARE are displayed in YRB. **(B,C,H)** t-SNAREs, [syntaxin (S186-S259), red; SNAP25 (L11-K83/G132-G204), green], and **(D–F)** the v-SNARE [synaptobravin (L26-K94), blue], are displayed in YRB. **(A,G)** The SNARE complex is shown in ribbon representation indicating the four-helix bundle (red, green, blue; PDB code 1sfc; Sutton et al., 1998). **(C–D)** Matching electrostatic interactions (E41-R161, K52-D172, K59-D179, and K85-D250) between synaptobrevin and syntaxin/SNAP25 are indicated by arrows. **(H–J)** Zoom into the matching electrostatic interactions in the SNARE complex, indicated by circles. **(K)** SNARE complex colored according to electrostatic potential.

We now set out to test the YRB scheme on a highly hydrophobic interface. A system in which hydrophobic interactions play a key role in complex formation is the SNARE family (Sutton et al., 1998). This complex is involved in membrane fusion of vesicles and contains two t-SNAREs, SNAP25, and syntaxin, and one v-SNARE, synaptobrevin (Sutton et al., 1998; Weber et al., 1998; Südhof and Rothman, 2009). The crystal structure reveals a hydrophobic groove build up by a 3-helix bundle [syntaxin and SNAP25, in which the hydrophobic string of the fourth helix (synptobrevin) is inserted to initiate membrane fusion (Supplementary Table 4)] (Figures 6A,G). The side of this interface contains a hydrogen bond network and six charged pairs. The complementary views of the interface between v-SNARE and t-SNAREs in YRB displayed the hydrophobic core (Figures 6B–F). As in the crystal structure, YRB shows that several charged groups match between the complementary views alongside the edge of this hydrophobic groove (Figures 6C–D; arrows). The zoom into the edge of the interface displays that the following charged pairs of synaptobrevin and syntaxin/SNAP25 (E41-R161, K52-D172, K59-D179, and K85-D250) match between opposite surfaces (Figures 6H–J; charged pairs circles). Together, YRB highlighting visualizes SNARE specificity by matching of functional groups, such as the electrostatic and hydrophobic interactions.

### YRB highlights the hydrophobic nucleotide-binding pocket in Hsp90

**Figure 7:**
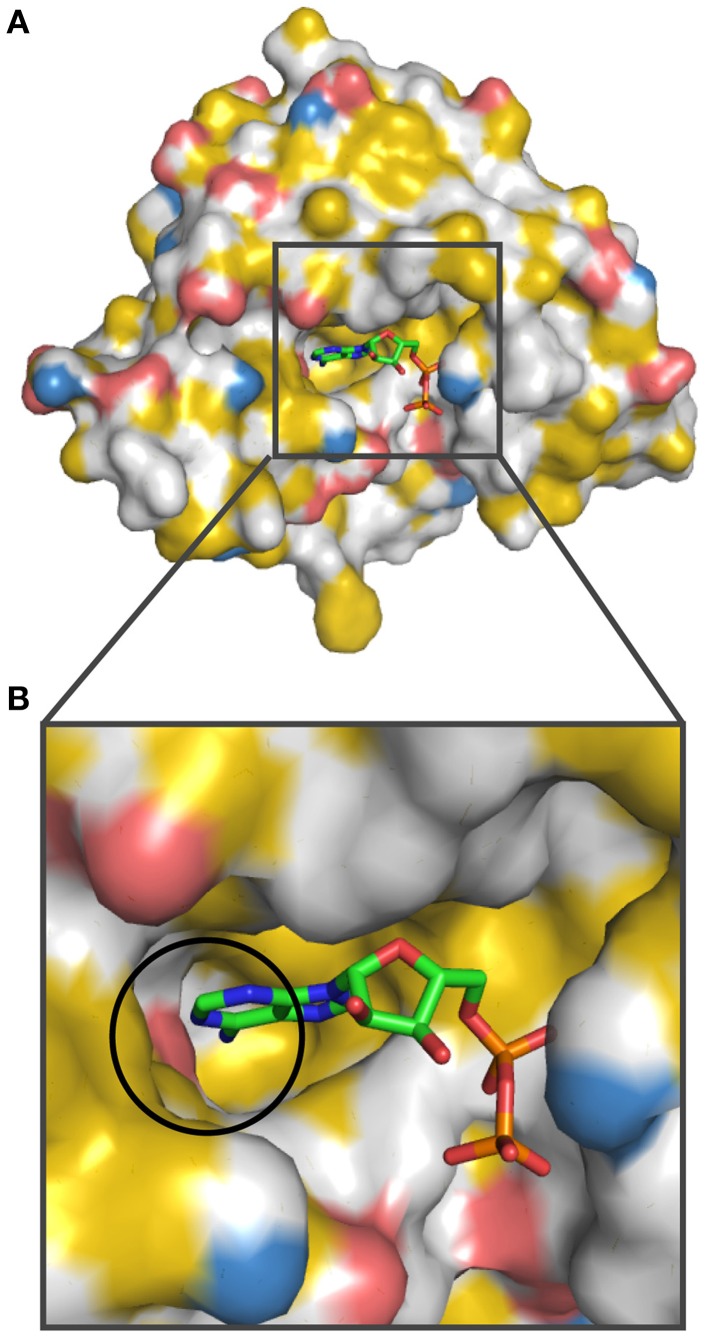
**Hsp90-nucleotide interaction in YRB colors**. The nucleotide-binding pocket in the Hsp90 N-terminal domain is displayed in YRB [human Hsp90-N (A2-E214) bound to ADP; PDB code 1am1 (Prodromou et al., 1997)]. **(A)** Overview of Hsp90 N-terminal domain and **(B)** a zoom into the nucleotide-binding pocket complexed with ADP. The interaction between Asp79 of Hsp90-N and the N-amino group of the adenine base is indicated by a circle. ADP is displayed according to CPK color scheme (carbon, green; oxygen, red; nitrogen, blue; phosphate, orange).

Next, we analyzed how pockets for small molecules are represented by YRB highlighting. Therefore, we analyzed the nucleotide binding pocket in Hsp90-N (Prodromou et al., 1997). The pocket, which binds to ATP and with even higher affinity to ADP, consists of a hydrophobic core, an extensive hydrogen-bonding network and most notably Asp79 co-ordinating the N-amino group of the adenine base. We colored the N-terminal domain of yeast Hsp90 bound to ADP according to the YRB scheme (Figure 7). The nucleotide-binding pocket in YRB colors displayed a mostly hydrophobic pocket, in which the interaction of Asp79 with the N-amino group of the adenine base of ADP is prominently visible (Figure 7B; circle). Thus, YRB visualizes the key determinants of the ATP pocket of Hsp90.

### A membrane protein with extended hydrophobic surface

**Figure 8:**
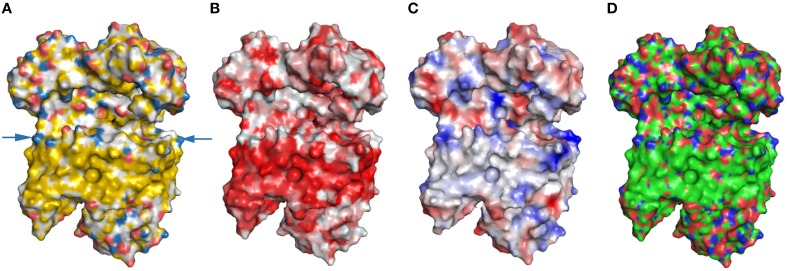
**Highlighting a transmembrane protein**. NADH:quinone oxidoreductase complex contains several domains (PDB code 4p6v; Weber et al., 1998). Na^+^NQR is highlighted either in **(A)** YRB, or **(B)** hydrophobic (red) to non-hydrophobic (white) gradient, or **(C)** according to electrostatic potential, or **(D)** the CPK coloring mode (carbon, green; oxygen, red; nitrogen, blue; phosphate, orange). A ring formed by positive charged head groups on the cytosolic side is marked by arrows.

Some of the most extensive hydrophobic surfaces are within membrane proteins. As membrane proteins need to interact with the lipid bilayer, they contain extended hydrophobic membrane domains. Previously, we have shown that YRB highlights hydrophobic contributions to surfaces. Therefore, we highlighted the NADH:quinone oxidoreductase complex in YRB. Na^+^-NQR is a membrane complex and facilitates Na^+^ translocation across the membrane (Steuber et al., 2015). YRB displays the hydrophobic membrane domain of the NADH:quinone oxidoreductase complex (Figure 8A). In addition, YRB reveals a positively charged ring at the cytoplasmic side of the transmembrane domain, that has not been described before for this complex. Thus, YRB displays the extensive hydrophobic membrane domain of the Na^+^-NQR and reveals a positively charged band at the cytoplasmic side of the transmembrane domain.

### YRB highlighting compared to alternative schemes to color protein surfaces

The YRB scheme combines three features that facilitate assessment of protein interaction interfaces: (i) YRB highlights surface properties at atomic level. (ii) YRB only colors the hydrocarbons with non-polar substitutions hydrophobic and distinguishes between hydrocarbons groups with polar and non-polar substitutions. (iii) YRB visualizes charged groups in interaction interfaces. As mentioned in the introduction there are alternative coloring schemes that represent properties of proteins. How does the YRB scheme compare to other highlighting schemes, in particular CPK coloring, the hydrophobicity scale and the electrostatic potential?

### YRB in comparison to CPK coloring

The CPK scheme colors per atom type, whereas the YRB scheme colors atoms based on functional properties (Corey and Pauling, 1953; Koltun, 1965). This means that YRB separates between oxygens and nitrogens in the backbone, in charged amino acids and in polar amino acids. Whereas, CPK does not distinguish between oxygen and nitrogen atoms that are either just polar or form ionic bridges. Distinguishing the charged groups from polar atoms would facilitate to identify the potential contributions of salt bridges to specificity in protein-protein interfaces. CPK is designed to distinguish between atom types, which is useful when analyzing molecular structures in stick mode. In contrast, YRB does not visualize polar interactions, which sometimes also may contribute to specificity. YRB, however, distinguishes between carbon atoms with polar and non-polar substitutions.

**Figure 9:**
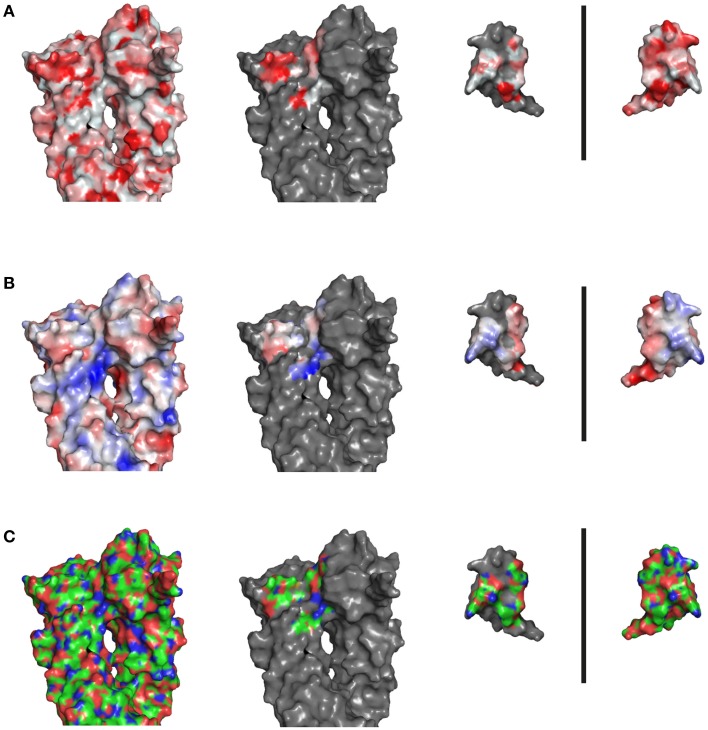
**Hsp90-p23 interfaces in hydrophobicity gradient, electrostatic potential and in CPK coloring**. Complex of yeast Hsp90 (A2-N676) and Sba1 (W12-A135), PDB code 2cg9 (Ali et al., 2006). **(A)** Hsp90/co-chaperone interfaces are shown in a hydrophobic gradient: non-hydrophobic white to hydrophobic amino acids red **(B)** in electrostatic potential and **(C)** in CPK coloring: carbon green, oxygen red, nitrogen blue, sulfate atoms yellow.

Although CPK and YRB both color at atomic level, they may differ in the potential to reveal specifics of protein interfaces. Therefore, we compared the Hsp90-p23 interface colored in YRB and CPK. CPK colors all carbons and therefore it also reveals the hydrophobic contributions of the Hsp90-p23 interface as in YRB. Only YRB distinguishes between hydrocarbons with polar and non-polar substitutions, in other words YRB does not color the carbons in the polar backbone as hydrophobic, among others (Figures 3–5, 9). Also, CPK coloring did not specifically reveal charged atoms. YRB explicitly highlighted only the hydrophobic and charged contributions, whereas the polar regions remain white. In this way, the hydrophobic contributions pop out in YRB. Besides, in CPK coloring the difference between polar atoms and charged atoms is not made. The Hsp90/co-chaperone interfaces in CPK colors do not stress the electrostatic interactions individually (Figure 9). In YRB highlighting the electrostatic contributions are individually colored and, therefore, reveal key determinants of protein-protein interfaces (Figures 3–5). Thus, CPK coloring does reveal the hydrophobic contributions, but as CPK does not make an exception between charged and polar atoms, it does not reveal specificity. Next, we wondered how CPK colors the hydrophobic membrane domain of the NADH:quinone oxidoreductase complex. CPK reveals the hydrophobic membrane domain and distinguishes the polar from the non-polar regions, but it does not reveal the charged ring of the NADH:quinone oxidoreductase complex visible in YRB coloring (Figures 8A,D). Together, both YRB and CPK highlighting provide insights into surface properties, and it depends on the research question which one of these complementary schemes is more suitable.

### YRB in comparison to the hydrophobicity gradient

We compared YRB and hydrophobicity highlighting (Eisenberg et al., 1984) in the Hsp90-p23 complex (Figures 4, 9A). The red hydrophobic regions of the Hsp90-p23 interface do not entirely match with the hydrophobic regions colored in yellow for YRB. Some parts of red hydrophobic regions do overlap with polar regions colored in white for YRB (Figures 4, 9). This is due to the hydrophobic gradient depicting some polar regions as if they were hydrophobic. Thus, YRB colors the hydrophobic regions more precisely than the hydrophobicity gradient does.

As mentioned above, YRB revealed the hydrophobic membrane domain of the NADH:quinone oxidoreductase complex and a charged ring at the cytoplasmic side of the transmembrane domain. Both highlighting schemes faithfully represent the hydrophobicity of the transmembrane segment (Figures 8A,B). In addition, however, YRB also visualizes a ring of positively charged head groups on the cytoplasmic side of the transmembrane domain (Figure 8A).

### YRB in comparison to electrostatic potential maps

The electrostatic potential map is a widely used coloring scheme that colors protein surface based on their overall charge distribution. As YRB colors the charged atoms separately, we wanted to know how YRB compares to the electrostatic potential map of the Hsp90-p23 interface. Therefore, we highlighted the Hsp90-p23 interface in the electrostatic potential map (Figure 9B). The electrostatic map of this interface displays the overall charge distribution, in which red indicates negative and blue positive potential. Overall, the electrostatic potential map of the p23 interface matches with the map of the Hsp90 interface. YRB, however, highlights two charged pairs in the interface (Figures 4C,D).

YRB highlighting revealed six charged pairs of the SNARE interface. Does the electrostatic potential maps also display these charged pairs of the SNARE interface? The electrostatic potential map of the SNARE complex displays the overall charge distribution at the edge of the interface between v-SNARE and t-SNAREs (Figure 6K). Whereas, the YRB highlighted interface gives the precise electrostatic interactions between the v-SNARE and t-SNAREs (Figure 6J). The same accounts for the positively charged ring of Na^+^NQR membrane complex (Figures 8A,C). Thus, the electrostatic potential map gives the overall charge distribution, whereas YRB provides the specific location of the charges within interaction interfaces.

## Discussion

YRB highlighting is based on properties of functional groups of amino acids. It simultaneously highlights two determinants of specificity in protein-protein interaction at atomic level, hydrophobicity and charge. For assessing hydrophobicity, we highlight all carbon atoms not bound to nitrogen or oxygen atoms. Simultaneously, we color all nitrogens and oxygens of the charged groups of arginine, lysine, aspartate, and glutamate. Here, we have shown that YRB reveals the two specificity determinants, hydrophobicity and charges, by the interfaces of Hsp90/co-chaperones and the SNARE complex.

**Figure 10:**
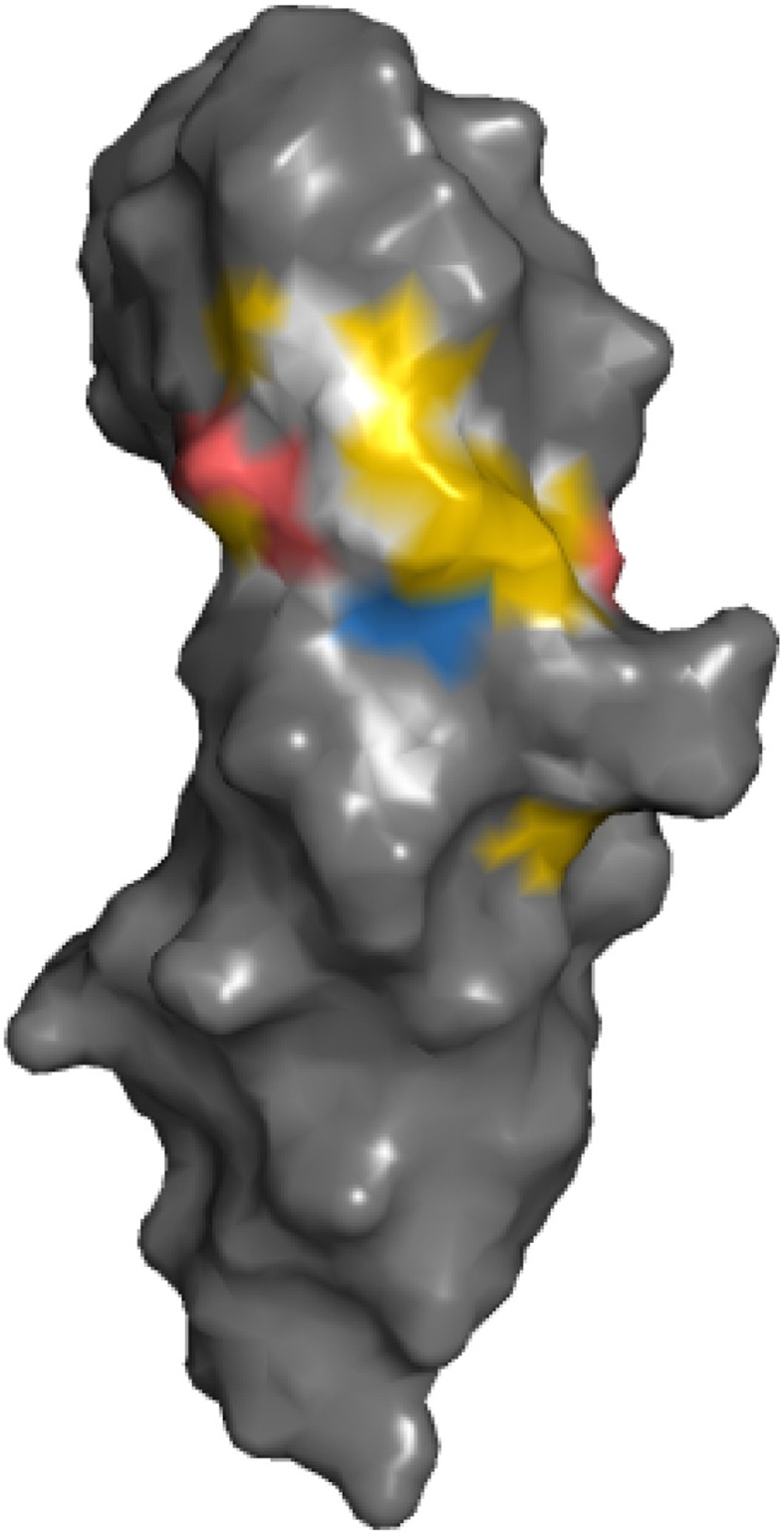
**YRB reveals the binding hot spot of the growth hormone receptor**. The extracellular domain of the growth hormone receptor (hGHbp; E32-P234), PDB code 3hhr (de Vos et al., 1992). The binding hot spot is determined by alanine substitutions of the residues in the interface (all residues with ΔΔG > 1 kcal/mol) (Clackson and Wells, 1995).

Highlighting of hydrophobic and charged atoms reflect that those residues are particularly important in protein-protein interfaces. Binding hot spots in general are enriched in tryptophan, tyrosine and arginine (Bogan and Thorn, 1998; Winter et al., 2012). Alanine scanning of the binding interface of a growth hormone-receptor interaction reveals that residues contributing to the hydrophobic interface are most important for affinity; further contributions are also made by several charged interactions while polar interactions are less important (Clackson and Wells, 1995). These observations are also reflected in the YRB scheme when coloring the binding surface of the growth hormone receptor according to the YRB scheme (Figure 10).

We should stress that the YRB scheme has intrinsic limitations that need to be taken into account when assessing protein-protein interactions. YRB visualizes structural models, and the quality of the representation depends on eventual limitations of the structural model. In particular, proteins are dynamic and may undergo local and even global conformational changes. Surface-exposed amino acids have a certain degree of freedom in solution, which is not represented in a static YRB colored protein model. When it comes to charges, YRB identifies salt bridges in protein complexes, but for assessing long-range electrostatic interactions the overall electrostatic potential map will in many cases be more suitable. We encourage any interested users to highlight protein models using different schemes, depending on the problem to be addressed.

Large hydrophobic and aromatic side chains also play an important role for the recognition by molecular chaperones, together with positively charged residues (Knoblauch et al., 1999; Patzelt et al., 2001; Rüdiger et al., 2001). The analysis of the substrate recognition site of the molecular chaperone Hsp90, however, makes it evident that it is also important to consider hydrophobic contributions of residues that are otherwise polar or even charged (Karagöz et al., 2014; Karagöz and Rüdiger, 2015). The YRB scheme takes this into account by highlighting proteins at atomic level, which visualizes all hydrophobic contributions at a glance.

We have shown that YRB visualizes complementarity of functional groups, such as hydrophobic and charged interactions, with a set of examples that represent different types of interaction including Hsp90/co-chaperone interfaces, the SNARE complex and Hsp90 nucleotide binding. The interfaces in YRB display matching of complementary functional groups between opposite surfaces, visualizing key specificity determinants (Figures 3–6). YRB is suitable for identifying charged pairs simultaneously with mapping hydrophobic interactions. Compared to alternative highlighting schemes, it visualizes information at a glance in one image, which would otherwise require combining several images (Figure 9).

YRB highlighting may be useful to identify binding interfaces that are determined by hydrophobic interactions, such as those of molecular chaperones and SNARE proteins. It provides an intuitive assessment of the potential of protein surfaces to interact with binding partners. YRB runs as a script in PyMOL and does not require programming experience.

## Author contributions

DH and IAEMvB wrote the YRB script. DH has analyzed the potential of the YRB script by analysing the interfaces mentioned in the article. SGDR conceived the work. DH, SGDR, and IAEMvB have contributed to writing the article. DH made all figures and tables. TML has contributed to both supervision of the project and discussions and has provided comments on the manuscript.

## Funding

SGDR was supported by Marie-Curie Actions of the 7th Framework programme of the EU [Innovative Doctoral Programme “ManiFold” (No. 317371) and Initial Training Network “WntsApp” (No. 608180)] and by the Internationale Stichting Alzheimer Onderzoek (ISAO; project “Chaperoning Tau Aggregation”; No. 14542).

### Conflict of interest statement

The authors declare that the research was conducted in the absence of any commercial or financial relationships that could be construed as a potential conflict of interest.
