# Supplementary material for: A script to highlight hydrophobicity and charge on protein surfaces
Source: Front Mol Biosci. 2015 Oct 13;2:56. doi: 10.3389/fmolb.2015.00056 (PMC4602141; doi:10.3389/fmolb.2015.00056)
Supplement: Supplementary file 1 [file Table1.DOCX]

|  | |  |  |  |
| --- | --- | --- | --- | --- |
| **Table 1: Interaction characteristics between amino acids of Hsp90-N and Cdc37 specified^a^** | | | | |
|  | **Hsp90-N** | **Cdc37** | **Interaction** | |
|  | M116  F120  A112  A107  A103  A110 | L165 M164  A204  L205 | $\left\{ \begin{aligned} a \\ a \\ a \\ a \\ a \\ a \end{aligned} \right.$Hydrophobic^b^ | |
|  | E33 | R167 | charged pair | |
|  | S36  Q119  S109  E106 | R167  D170, R167, R166, Q208  K202  A204 | H-bond  H-bond  H-bond  H-bond | |

1. The interface according to Roe et al., 2004
2. Hydrophobic residues typically interact with several hydrophobic residues and therefore are listed as patches.

| **Table 2: Interaction characteristics between amino acids of Hsp90 and p23 specified^a^** | | | |
| --- | --- | --- | --- |
| **patch** | **Hsp90** | **p23** | **Interaction** |
| 1^b^ | T13, S17, I20, N21, N151-S155 | A33, D34  A85-Y90 | hydrophobic and hydrogen bonds |
| 2^c^ | The surface of the lid: F104-L108,  G111-V114, I117, Q119  D113 | Q14-S16,  K113-V116, K118  K113 | hydrophobic and hydrogen bonds  Potential charge pair |
| 3^d^ | K27  K387  L315, P375, I388, V391 | D122  F121, D122, W124  F121, W124 | charged pair  hydrogen bonds  hydrophobic |

1. Interface according to Ali et al., 2006. Listed are residues of the Hsp90-p23 interface within 5Å of the other protein.
2. The lower resolution of the crystal limits further specifications.

| **Table 3: Interaction characteristics between amino acids of Hsp90-M and Aha1 specified^a^** | | | |
| --- | --- | --- | --- |
|  | **Hsp90-M** | **Aha1** | **Interaction** |
|  | V391, I388, L315 T433 | F100, L66, I64  P96 | hydrophobic  hydrophobic |
|  | Q314 | S65-I64 | hydrogen bond |
|  | K398  K387  K394  K390 K469  K514  E515 | D101  D53  E97  D68  E122  D110  R128 | charged pair  charged pair  charged pair  charged pair  charged pair  charged pair  charged pair |

1. Interface according to Meyer et al., 2004

| **Table 4: Interaction characteristics between amino acids of syntaxin/SNAP25 and synaptobrevin** | | | |
| --- | --- | --- | --- |
| **Syntaxin / SNAP25** | | **Synaptobrevin** | **Interaction** |
| R232  D250 | R161  D172  D179  D186 | E41  K52  K59  D64  R66  K85 | charged pair  charged pair  charged pair  charged pair  charged pair  charged pair |
| I202  L205  I209  L212  M215  F216  M219  L222  V223  M229  I230  I233  V237  A240  Y243  V244  A247  T251  A254  Y257 | L50  L57  M64  I67  M71  A74  L78  L81  F133  I134  L150  I157  L160  A164  L165  M167  I171  I178  I181  M182  A185,  I192  A195 | T26  L32  T35  V39  V42  V43  I45  M46  V50  V53  L60  S61  L63  A67  A69  L70  A74  F77  A81  A82  L84  Y88  W89 | $\left\{ \begin{aligned} a \\ a \\ a \\ a \\ a \\ a \\ a \\ a \\ a \\ a \\ a \\ a \\ a \\ a \\ a \\ a \\ a \\ a \\ a \\ a \\ a \\ a \end{aligned} \right.$Hydrophobic |
| K204, S208  Q226  S259 | I134  G122  Q174, Q53  N196  M202, L203 | N29  R31  Q36  R56, D57  Q76, S80  K87  N92 | Hydrogen bonds |

1. Interface determined following these criterea: Hydrophobic <4.5Å, Hydrogen bond, 2.5-3.2Å.
